# Ablative vs. Non-Ablative Radiotherapy in Palliating Locally Advanced Pancreatic Cancer: A Single Institution Experience and a Systematic Review of the Literature

**DOI:** 10.3390/cancers15113016

**Published:** 2023-06-01

**Authors:** Randa Kamel, Kristopher Dennis, Janice Doody, Jason Pantarotto

**Affiliations:** 1 Department of Radiation Oncology, UZ Brussel, Vrije Universiteit Brussel, Laarbeeklaan 101, 1090 Brussel, Belgium; 2 Department of Radiation Oncology, The Ottawa Hospital, Smyth Road 501, Ottawa, ON K1H 8L6, Canada

**Keywords:** locally advanced pancreatic cancer, stereotactic body radiotherapy, stereotactic ablative radiotherapy, palliative radiation therapy, systematic review of the literature, retrospective single institution cohort study

## Abstract

**Simple Summary:**

The very poor prognosis and the absence of radical treatment options in cases of unresectable “locally advanced pancreatic cancer (LAPC)” focus its management on palliation. An “overall survival (OS)” benefit has been shown with new systemic therapy agents; however, not all patients are good candidates for systemic treatments at diagnosis, and LAPC is often associated with a high symptom burden that greatly impacts patients’ quality of life. Studying dose optimization of radiation as an option for local progression cessation is therefore necessary. Research in this setting as opposed to other treatment sites is under-represented in the literature and the guidelines are based on very scarce data. We aim to present the outcomes of “stereotactic body radiotherapy (SBRT)” (ablative radiotherapy) vs. non-ablative radiotherapy through our patient population and the available data in the literature.

**Abstract:**

We studied the dose–local control (LC) relationship in ablative vs. non-ablative radiotherapy in a non-radical treatment setting of “locally advanced pancreatic cancer (LAPC)” by comparing our patients (n = 89) treated with SBRT on the CyberKnife unit vs. conventional radiation between January 2005 and January 2021, and by reviewing the literature. A systematic search was performed leveraging Medline for references on SBRT use in pancreatic cancer without date terms or language restrictions. A total of 3702 references were identified and the search was then repeated in Embase and the Cochrane database. Ultimately, 12 studies were eligible for inclusion, which either compared SBRT to conventional radiation, or SBRT use in dose escalation for primary LAPC in a non-neoadjuvant setting. Our cohort’s median overall survival was 152 days (CI 95%, 118–185); including 371 days (CI 95%, 230–511) vs. 126 days (CI 95%, 90–161) favoring SBRT, *p* = 0.004. The median time to local progression was 170 days (48–923) for SBRT vs. 107 days (27–489) for the non-ablative group. In our SBRT patients, no local progressions were seen with BED_10_ > 60 Gy. Even when palliating LAPC, SBRT should be considered as an alternative to conventional radiation, especially in patients with a low disease burden. BED_10_ ≥ 60–70 Gy offers better local control without increasing toxicity rates. Less local progression may provide a better quality of life to those patients who already have a short life expectancy.

## 1. Introduction

While surgery is the current sole radical treatment option in pancreatic cancer, only a minority of patients present with resectable disease at the time of diagnosis. Studies addressing newer chemotherapy regimens show a survival benefit for “locally advanced pancreatic cancer (LAPC)” [1,2]; however, local progression remains problematic and often presents with a high symptom burden. The main goal of “radiotherapy (RT)” in this setting is to prevent or delay local progression.

The role of RT in unresectable pancreatic cancer has always been controversial regarding its relatively radioresistant nature and the modest 1-year “local control (LC)” rates that “conventionally fractionated radiotherapy (CFRT)” offers. While this role is currently evolving due to the advancement of RT techniques allowing for higher dose delivery and expansion of the therapeutic window [3], the guidelines on RT dose as a monotherapy in LAPC are based on very scarce data [4,5].

“Stereotactic body radiotherapy (SBRT)” in fewer fractions is more convenient to patients in terms of logistics than CFRT and can decrease the elapsed time to undergo another treatment modality. Moreover, SBRT yields a higher “biologically effective dose (BED)” which could potentially allow for better LC rates. In 2004, Koong et al. [6] was the first to show feasibility of using SBRT in LAPC. In this phase I dose escalation study, doses up to 25 Gy in a single fraction were delivered and the 1-year local control rate was 100% in the patient subgroup who received 25 Gy, without grade 3 or higher acute gastrointestinal toxicity. Since then, there has been an increased interest in using SBRT in LAPC; however, this is with few prospective data and no phase III trials comparing its use to CFRT.

At The Ottawa Hospital, we previously studied the outcomes of using different dose schemes of non-ablative hypo-fractionated RT with a palliative intent to the primary unresectable LAPC, treated between 2005 and 2019. The results showed a survival benefit for the group of patients who were treated within the higher dose range of ≥30–40 Gy in 10–15 fractions, however, local progressions were common. (Manuscript submitted, under review). We aimed here to study the difference in outcomes between SBRT and conventional RT and observe if higher biologically effective doses could defer local progression without substantially increasing toxicity. We compared our higher dose range palliative patients to those who received SBRT in a non-curative setting, hypothesizing that performance status and the disease extent in these two groups were likely not that different and that local control is probably better with SBRT. We then conducted a systematic review of the literature to identify studies that similarly compared different dose arms in LAPC in a non-curative, non-neoadjuvant setting.

## 2. Materials and Methods

This is a single-institution, ethics approved, retrospective study carried out at a large Canadian cancer center, the main treatment facility for approximately1.4 million people. Electronic records were reviewed for all patients who received SBRT to the pancreas on a CyberKnife robotic radiosurgery unit (Accuray Incorporated., Sunnyvale, CA, USA) from September 2010 to January 2021. In the non-ablative RT group, all patients who received ≥30–40 Gy in 10–15 fractions to the pancreas between 1 January 2005 and 31 December 2019 were included. In the primary analysis, primary pancreatic irradiations and local recurrences from both groups were included. All included patients had a biopsy-proven diagnosis. Patients who received non-ablative RT doses of <30 Gy were excluded because they had worse performance statuses and experienced more anti-symptomatic RT (e.g., bleeding due to invasion of nearby organs, etc.). Patients who had concurrent chemotherapy during RT were also excluded. The potential end for follow-ups was 1 February 2021.

Outcomes of interest included demographics, clinical, and RT details. Baseline diagnostic images and reports were reviewed to assign TNM values according to the AJCC 8th edition system for pancreatic ductal adenocarcinomas. Descriptive statistics summarized data including medians and ranges for continuous variables, as well as frequencies and proportions for categorical variables. The Kaplan–Meier method was used for time-to-event analyses on SPSS statistical software; version 28.0.1.0 (142).

Overall survival (OS) was calculated from the start of the RT course until death from any cause, and patients were censored at the last follow-up if alive. In-field progression was calculated from the start of the RT course until radiographic confirmation of local progression, either on computed tomography or magnetic resonance imaging.

**Figure 1:**
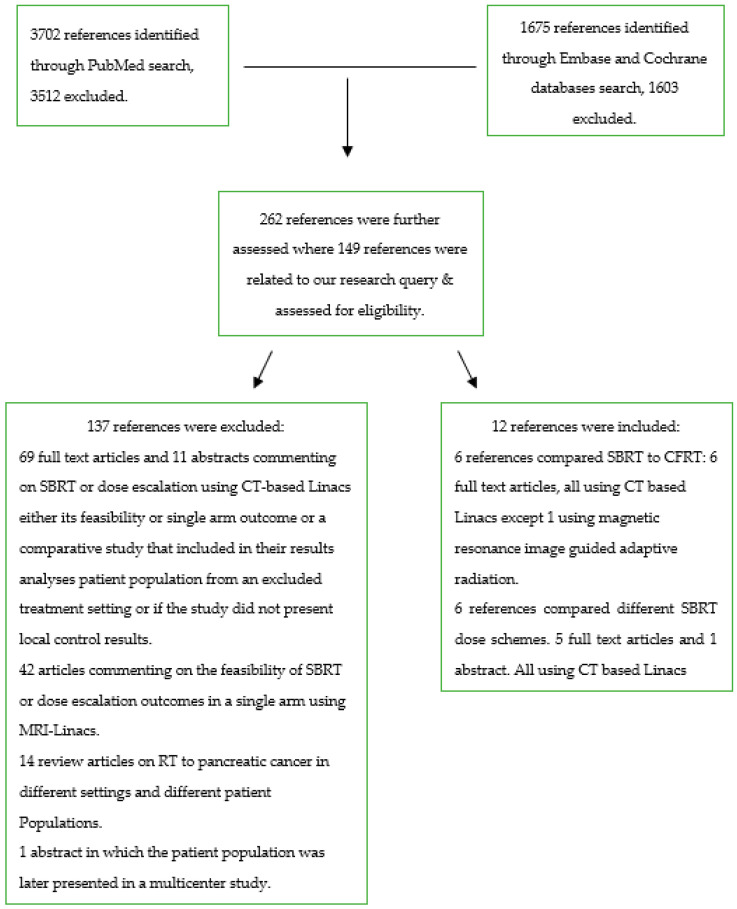
Flow diagram of literature search.

Then, a systematic search was conducted leveraging Medline via PubMed for all references reporting on the use of SBRT or ablative radiation in pancreatic cancer without date terms or language restrictions, for which the search flow diagram is described in Figure 1. From Medline, 3702 references were identified and screened, from which 190 references were deemed relevant. Then, the search was repeated in Embase and the Cochrane database for systematic reviews, including the Cochrane central trials database where 1675 references were screened and relevant references were identified. After removing duplicates, an extra 72 references were added to a total of 262 references. Following further screening, 149 articles reporting on the clinical use and outcomes of SBRT in pancreatic cancer were studied for eligibility, including their reference lists. The databanks were last checked on 22 September 2022.

The eligibility criteria included studies which compared different RT dose scheme outcomes, either by comparing SBRT to CFRT, or by comparing different SBRT doses to report on dose effects on local control in primary LAPC in a non-neoadjuvant setting.

Ultimately, 12 studies were eligible for our review: 11 full-text articles and 1 abstract. Since no randomized trials were conducted in this setting, the risk of bias assessment was performed using the risk of bias in non-randomized studies of intervention (ROBINS-I) tool [7]. Data were abstracted and categorized for comparison. The main points of interest were the full radiotherapy dose data; the calculation of the BED_10_; the 1-year LC (or 2-year if the 1-year data were not reported); the recurrence rate; the free from local progression rate; and the local progression-free survival. Other points of interest were the overall survival rate and chemotherapy administration. Table 1 summarizes the data extracted from the eligible studies.

## 3. Results

In our cohort, a total of 89 patients were analyzed with the full patients’ characteristics summarized in Table 2. A total of 74 patients received non-ablative hypo-fractionated RT and 15 patients received SBRT. The biologically effective dose was calculated for an α/β of 10 Gy (BED_10_). The non-ablative RT group received a median dose of 30 Gy in 10 fractions (range: ≥30–40 Gy in 10–15 fractions) and a median BED_10_ of 39 Gy (range: 39–56 Gy), while the SBRT group received a median dose of 21 Gy in three fractions (range: 21–50 Gy in 3–5 fractions) and a median BED_10_ of 35.7 Gy (range: 35.7–100 Gy). The full RT data are described in Table 3.

In the non-ablative RT group, 67 patients were treated for their primary tumor, while seven patients were treated for local recurrences post resection. In the SBRT group, 12 patients were treated for their primary tumor, while three patients were treated for recurrences (two patients were post resection and one patient was post prior chemoradiotherapy, where the prior RT regimen was conventionally delivered to a dose of 50.4 Gy). In addition, five SBRT patients received elective nodal irradiation post SBRT of 45 Gy, delivered using intensity modulated radiotherapy (IMRT).

The median age for the whole cohort was 68 years (range 44–83). There were slightly more male (n = 46/89, 52%) than female (n = 43/89, 48%) patients. All patients in the SBRT group were ECOG 1 at the RT start, while half of the patients in the non-ablative RT group were ECOG 1 (n = 36/74), three patients were ECOG 0, and the rest were ECOG 2–3. Only one of the non-ablative RT patients who began the treatment as outpatients required hospitalization prior to the RT completion, while all patients from the SBRT group started and ended the RT as outpatients. Most primary tumors were T4 (n = 52/89, 58.5%) and most of them were located in the pancreatic head (n = 47/89, 53%). Almost half of the non-ablative RT patients were node-positive and were metastatic at the RT start, while all SBRT patients were node-negative and non-metastatic at the RT start. Of the whole cohort, 42% (n = 37/89) of the patients were stented at the RT start. A total of 44% (n = 39/89) of patients received neoadjuvant systemic therapy, mainly Gemcitabine and FOLFIRINOX. Only one patient received a second course of antihemorrhagic RT; this patient belonged to the non-ablative group, and only information from the first RT course was used for this analysis. Most patients in the non-ablative RT group were treated with 3D conformal RT (n = 41/74, 55%) or IMRT/VMAT (n = 28/74), from which only 10% (n = 8/74) from the non-ablative group were simulated with four-dimensional CT. All SBRT patients had endoscopically or percutaneously placed fiducials and four-dimensional CT at simulation with intravenous contrast.

### 3.1. Overall Survival

**Figure 2:**
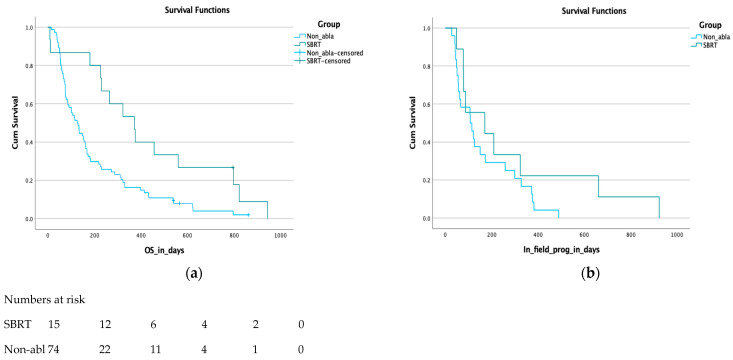
(**a**) Overall survival in days; (**b**) days from RT to local progression.

The median OS of the whole cohort was 152 days (5 months) from the RT start (CI 95%, 118–185 days). For the SBRT group, the median OS was 371 days (12 months, CI 95%, 230–511 days) vs. 126 days (4 months) for the non-ablative RT group (CI 95%, 90–161 days). On the log rank test, there was a significant survival benefit favoring the SBRT group, *p* = 0.004 (see Figure 2a).

### 3.2. Subgroup Analysis

**Figure 3:**
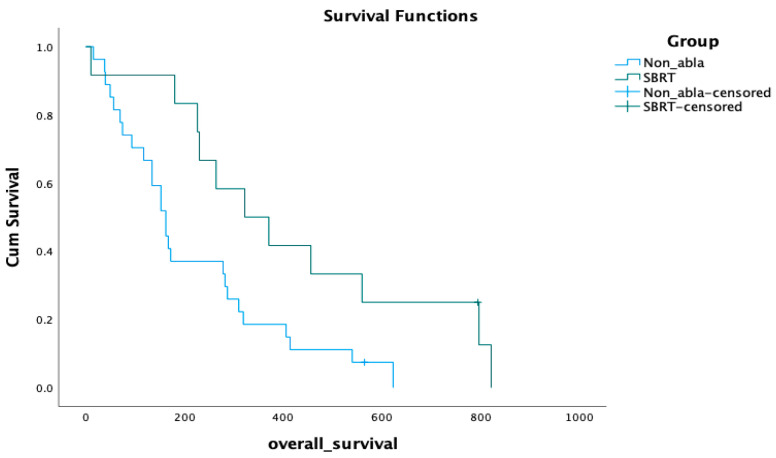
Overall survival in subgroup analysis.

We performed a subgroup analysis restricted to the non-metastatic patients who received RT to their primary tumors; these were 27 patients from the non-ablative RT group vs. 12 patients from the SBRT group. The overall survival was 322 days (11 months, CI 95%, 140–503 days) for the SBRT group vs. 162 days (5.5 months, CI 95%, 126–197 days) for the non-ablative RT group. The survival benefit remained significant favoring the SBRT group, *p* = 0.018 (see Figure 3).

### 3.3. Local Progression

A total of 24/74 patients in the non-ablative RT group experienced local progression, with a median of 107 days (>3 months, range: 27–489 days) from the RT start. While 9/15 SBRT patients recurred locally with a median of 170 days (>6 months, range: 48–923 days). This difference was non-statistically significant (*p* = 0.1, see Figure 2b). No local progression was seen in patients who received SBRT with a dose >30 Gy in three fractions (BED_10_ > 60 Gy).

### 3.4. Systematic Review Data

Six out of twelve eligible studies compared dose escalation between SBRT arms. In five studies, a significant LC benefit was found, favoring high dose groups. Pollom et al. [8], Toesca et al. [9], Zhu et al. [10], and Mazzola et al. [12] showed statistically better local control rates using EBD_10_ doses of >70 Gy. Arcelli et al. [13] compared BED_10_ ≥ 48 Gy vs. less, and a significant local control benefit was still found favoring the higher dose group. One study by Abi Jaoude et al. [11] showed no benefit when comparing BED_10_ ≥ 72 Gy vs. less. Three studies showed an OS benefit for the higher dose groups, one study did not provide information on the OS, and two studies found the OS was non-statistically significant. The remaining six studies compared CFRT to SBRT; however, in all cases, the doses had overlapping BED_10_ ranges. Only two out of six studies showed a statistically significant LC benefit favoring the SBRT group, seen in Lin et al. [18] and Arcelli et al. [15].

## 4. Discussion

In this paper, we questioned the feasibility of using SBRT as an alternative to conventional radiation when treating LAPC that is non amenable to local resection and where treatment is focused on palliation. In these cases, the decision on RT dose and technique is usually based on the treating physician’s intuition rather than evidence-based guidelines. We assumed that SBRT could be more convenient due to less visits and could provide a better local control. We presented the outcomes of our institution, together with the available data in the literature in this setting, while acknowledging the limitations of this retrospectively collected data.

In our cohort, both groups had similar demographics; however, patients in the SBRT group had less disease burden when they started treatment and were all non-metastatic, while more than half the patients in the conventional group were metastatic at the RT start. This can explain their better OS in the primary analysis. This survival benefit was nevertheless still significant (*p* = 0.018) in the subgroup analysis restricted to the primary irradiation of non-metastatic patients from each group. Looking at local progression, the SBRT patients showed more time to local progression than the conventional group, but this was not statistically significant (*p* = 0.1) which is likely explained by the overlapping median BED_10_ doses between both arms. We noticed, however, that all patients who received SBRT doses higher than 30 Gy in three fractions (BED_10_ > 60 Gy) did not experience local progression. SBRT was generally well tolerated and no patients needed hospitalization during treatment, but the toxicity data was not prospectively documented, limiting its interpretation. The systematic review data showed no significant OS or PFS difference when SBRT was compared to CFRT when similar BED_10_ doses were used in the compared arms. On the other hand, studies that compared different SBRT dose arms showed a better PFS when BED_10_ dose exceeded 70 Gy.

Over the past two decades, many single-institution retrospective studies have shown the feasibility of delivering SBRT in LAPC in different treatment settings with acceptable toxicity profiles [20]. Vornhülz et al. and Buwenge et al. [21,22] favored SBRT at reducing pain and quality-of-life improvement, even in elderly patients with poor performance status as studied by Rosati et al. and Ciabatti et al. [23,24]. Similarly, several studies compared the use of SBRT to CFRT where an OS benefit favoring SBRT was shown; however, most data are retrospective and included small patient groups [25,26,27,28,29,30]. In 2020, Mahadevan et al. [31] reviewed and analyzed data from 39 studies on the use of hypofractionated SBRT for pancreatic cancer in various clinical scenarios (e.g., preoperative [neoadjuvant], borderline resectable, and LAPC) and concluded that, in three-fraction equivalents, doses more than 28 Gy (BED_10_ > 54 Gy) resulted in better local control. They mentioned that Grade 3 toxicities associated with doses beyond 36 Gy in three fractions (BED_10_: 79.2 Gy) outweigh any potential benefits. Reddy et al. [32] also concluded inferior local control in local pancreatic cancer recurrences with BED_10_ < 54.8 Gy.

Zhu et al. [33] concluded better tumor response in advanced or medically inoperable pancreatic cancer with BED_10_ ≥ 60 Gy with significantly less toxicity rates when the BED_10_ dose did not exceed 70 Gy. Goldsmith et al. [34] recommended BED_10_ > 70 Gy with caution to the duodenum, especially with large treatment target volumes. Interestingly, Jolissant et al. [35] from Memorial Sloan Kettering Cancer Center retrospectively compared outcomes of ablative radiation after induction chemotherapy for T4 localized pancreatic cancer or tumor encasement of the big vessels vs. surgical resection. Patients were irradiated to BED_10_ doses > 97 Gy in hyperfractionated schemes of 3–4.5 Gy/fraction and had similar locoregional control rates as surgery, with similar major adverse gastrointestinal events in the two groups at 25%.

The above suggests that higher SBRT doses probably offer better survival, as well as local control rates with acceptable toxicity when BED_10_ did not exceed 70 Gy.

## 5. Conclusions

Even in a non-curative setting, SBRT can be considered as an alternative to conventionally fractionated RT in LAPC; especially in patients with a low disease burden. Moderately higher BED_10_ doses of ≥60–70 Gy offer better local control with acceptable toxicity rates. Less local progression can translate into a better quality of life to that subgroup of patients who already have a short life expectancy. Prospective data over toxicity using higher SBRT doses in LAPC is needed.

## Figures and Tables

**Table 1:** Data extracted from eligible studies.

Author, Year	Study Description and Total No. of Patients	Treatment Arms: Dose and No. of Fractions (fx)	BED_10_	Chemotherapy Received	Median Follow-Up	LC Results	Overall Survival	Risk of Bias According to ROBINS-I Tool
**Pollom, 2014 [8]**	Retrospective single-institution study, 167 patients	25 Gy in 1 “fraction (fx)”vs. median of 33 Gy (25–45 Gy) in 5 fx	87.5 Gy vs. median 54.8 Gy (37.5–85.5 Gy)	Yes, (neo)adjuvant	7.9 months	1-year local recurrence rate 9.5 vs. 11.7%, *p* = 0.8	Median 13.6 months; 30.8% vs. 34.9% at 1-year; no difference	Moderate
**Toesca, 2020 [9]**	Retrospective single-institution study, 149 patients	≥40 Gy in 5 fx vs. <40 Gy	≥72 Gy vs. less	Yes, neoadjuvant	15 months	Median PFS 13 vs. 10 months, *p* = 0.007; 1-year PFS 57% vs. 36%	Median 16 months; 82% vs. 57% at 1 year; statistically significant	Moderate
**Zhu, 2020 [10]**	Retrospective multicenter study, 972 patients	42 Gy in 5–8 fx (40–49.6 Gy) vs. 37 Gy in 5–8 fx (36–40.8 Gy)	74.6 Gy (range: 71.4–88.3 Gy) vs. 64.4 Gy (60.2–69.4 Gy)	Yes, adjuvant	20.2 months	Median PFS 15.4 vs. 13.3 months, *p* < 0.001	Median OS 20.3 vs. 18.2 months, *p* < 0.001	Moderate
**Abi Jaoude, 2022 [11]**	Retrospective single-institution study, 89 patients	≥40 Gy vs. <40 Gy in 5 fx	≥72 Gy vs. less	No information	No information	16.7 vs. 30.4% at 1 year, not statistically significant	No information	Not enough information for a full assessment
**Mazzola, 2018 [12]**	Prospective single-institution study, 33 patients	42–45 Gy in 6 fx vs. 36 Gy in 6 fx	>70 Gy vs. less	Yes	18 months	Overall, 81% at 1 year	Whole group 75% at 1-year; no statistically significant survival benefit	Low
**Arcelli, 2020 [13]**	Retrospective multicenter study, 56 patients	≥30 Gy with a median of 6 Gy per fx vs. <30 Gy	≥48 Gy vs. less	Yes, (neo)adjuvant	15 months	Better LC for BED ≥ 48 Gy and ≥6 Gy dose per fraction; *p* = 0.045 and *p* = 0.003, respectively	Median OS 20 vs. 15 months, *p* = 0.042	Moderate
**Rudra, 2019 [14]**	Retrospective multicenter study using ViewRay MRIdian, 44 patients	40–55 Gy in 25–28 fx vs. 30–35 Gy in 5 fx vs. 40–52 Gy in 5 fx vs. 50–67.5 Gy in 10–15 fx	Median 55.5 Gy vs. 55.8 Gy vs. 77.6 Gy vs. 82.7 Gy (divided in 2 groups > 70 Gy vs. less)	Yes, (neo)adjuvant	17 months	77 vs. 57% at 2 years, *p* = 0.15	49% vs. 30% at 2 years, *p* = 0.03 favoring high dose group	Moderate
**Arcelli, 2020 [15]**	Retrospective multicenter case control study, 80 patients	Median: 30 Gy in 6 Gy/fx vs. 50.4 Gy in 1.8 Gy/fx	Median 48 Gy vs. 59.4 Gy	Yes (neo)adjuvant	15 months	Median LC 22 vs. 16 months; 1-year LC 80.4 vs. 53%, *p* = 0.017	No difference	Moderate
**Shin, 2022 [16]**	Retrospective single-institution study, 161 patients	Median 28 Gy in 4 fx (24–36 Gy) vs. median 54 Gy in 1.8–2 Gy/fx (40–59.4 Gy)	Median 47.6 Gy (38.4–68.4 Gy) vs. median 64.8 Gy (48–70 Gy)	Yes, (neo)adjuvant	15.5 months	Free from local progression 77.2 vs. 87.1%; *p* = 0.691 at 1-year	66.7 vs. 80% at 1-year	Moderate to serious
**Park, 2017 [17]**	Retrospective single-institution study, 270 patients	30–33 Gy in 5 fx vs. 45–56 Gy in 25–28 fx	48–54.7 Gy vs. 53.1–67.2 Gy	Yes, (neo)adjuvant	12.9 months	34.4 vs. 30.2% at 1 year; not significant	Median 15.7 months, 56.2% vs. 59.6% at 1 year, *p* = 0.75	Moderate
**Lin, 2015 [18]**	Retrospective single-institution study, 41 patients	35–45 Gy in 7–9 Gy/fx vs. 45–50 Gy in 1.8–2 Gy/fx	59.5–85.5 Gy vs. 53.1–60 Gy	Yes, adjuvant in CFRT group	16 months	Significantly better LC favoring SBRT, *p* = 0.004	80 vs. 70.7% at 1 year, *p* = 0.127	Moderate to serious
**Abi Jaoude, 2021 [19]**	Retrospective single-institution study, 104 patients	Median 36 Gy (25–55 Gy) in 5 fx vs. median 50.4 Gy (50–50.4 Gy) in 25–28 fx	Median 61.9 Gy (37.5–115.5 Gy) vs. median 59.4 Gy (59.4–60 Gy)	Yes, neoadjuvant	22 months	PFS 16.1 vs. 12.3 months, *p* = 0.81	Median 29.6 vs. 24.1 months, *p* = 0.18	Moderate

**Table 2:** Patients’ characteristics.

Characteristics	Non-Ablative RT Group	SBRT Group
Number	N = 74	N = 15
**Median Age**	67 years (44–89)	69 years (53–83)
**Gender**		
Male	38	8
Female	36	7
**Performance Status**		
ECOG 0	3	0
ECOG 1	36	15
ECOG 2	20	0
ECOG 3	15	0
ECOG 4	0	0
**Hospitalization Status**		
Inpatient	11	0
Outpatient	63	15
**TNM**		
T1	0	2
T2	2	1
T3	11	3
T3/4	12	0
T4	46	6
Tx	3	5
N0	33	15
N1	26	0
N2	6	0
Nx	9	0
M0	31	15
M1	43	0
Mx	0	0
**Tumor Location**		
Head	39	8
Neck	3	1
Uncinate	0	1
Body	16	5
Tail	16	0
**Histology**		
Adenocarcinoma	70	15
Neuroendocrine carcinoma	2	0
Non-small cell carcinoma	1	0
Squamous cell carcinoma	1	0
**Stents In-Situ at RT start**		
Yes	30	7
No	44	8
**Prior Systemic Therapy**		
Yes	30	9
No	44	6

**Table 3:** Radiotherapy data.

	Total Dose Received	Number of Fractions	BED_10_	n	(% of 89 Patients)
**Non-ablative RT Group (n = 74)**	30 Gy	10	39 Gy	54	61%
	34 Gy	15	41.7 Gy	1	1%
	35 Gy	10	47.2 Gy	6	7%
	36 Gy	15	44.6 Gy	2	2%
	40 Gy	10	56 Gy	1	1%
	40 Gy	15	50.6 Gy	10	11%
**SBRT Group (n = 15)**	21 Gy	3	35.7 Gy	8	9%
	24 Gy	3	43.2 Gy	1	1%
	30 Gy	3	60 Gy	3	3.5%
	40 Gy	5	72 Gy	2	2%
	50 Gy	5	100 Gy	1	1%

## Data Availability

Data are available upon reasonable request to the corresponding author.
